# Granuloma annulare treated with ivermectin

**DOI:** 10.1016/j.jdcr.2026.03.020

**Published:** 2026-03-19

**Authors:** Anish Singh, Nelly Garza, Angela Bowers

**Affiliations:** Southlake Dermatology, Southlake, Texas

**Keywords:** granuloma annulare, ivermectin, novel, soolantra

## Introduction

Granuloma annulare (GA) is a benign inflammatory dermatosis that classically presents with annular groups of skin-colored to erythematous papules and plaques, often without significant epidermal change.^1^ The localized form is most common, frequently involving the dorsal hands or feet, while less common variants include generalized, subcutaneous, perforating, and atypical forms. Histologically, GA demonstrates characteristic features such as palisading granulomas or interstitial histiocytic infiltrates within the dermis, central degeneration (necrobiotic) of collagen, prominent mucin deposition, and a lymphohistiocytic inflammatory infiltrate; multinucleated giant cells are often present.^2^

GA’s precise etiology remains unknown. Recent gene expression profiling has revealed upregulation of Th2- and Janus kinase-/STAT pathways in GA lesions, supporting the notion that cytokine-mediated inflammation plays a central role.^3^ GA is also associated with systemic conditions such as diabetes, thyroid disease, and hyperlipidemia, and can be triggered by infections or other environmental stimuli, consistent with immune activation to an unidentified antigen.^4^

Localized GA is usually asymptomatic and self-limited, often resolving within 1 to 2 years; however, some cases persist, becoming chronic and refractory to standard therapies, justifying alternative treatment approaches.^1^ Treatment for localized disease is generally not required unless for cosmetic or symptomatic reasons; first-line therapies include topical or intralesional corticosteroids. For more extensive or recalcitrant disease, systemic immunomodulatory or immunosuppressive agents are sometimes used, though no therapy is FDA-approved specifically for GA, and supporting evidence is sparse, largely limited to case reports, small series, and retrospective reviews.^2^

Ivermectin, a macrocyclic lactone with antiparasitic and anti-inflammatory properties, has gained wide use in dermatology, particularly for rosacea. Randomized controlled trials have demonstrated that topical ivermectin 1% cream applied once daily is effective in reducing inflammatory lesion counts and improving global assessment scores, with a favorable tolerability profile.^5^ Its mechanism appears to be dual: decreasing Demodex mite density while downregulating proinflammatory mediators such as IL-8, TNF-α, TLR4, LL-37, and HBD3 in lesional skin.^6^ Pharmacokinetic studies show minimal systemic absorption with long-term use, and adverse effects are generally mild and localized. These antiparasitic and anti-inflammatory actions make ivermectin a versatile dermatologic therapy, though its role in GA has not been reported. Here, we present a case of GA showing a dramatic response to topical ivermectin after a 12-week course of therapy.

## Case report

A 66-year-old woman with a history of diabetes mellitus presented with a 7-year history of persistent, localized, and pruritic skin lesions involving the dorsal aspect of the right foot (2.4 cm) and the right calf (1.8 cm), which remained stable in size and distribution without documented flares or spontaneous resolution. On physical examination, a smooth, firm annular erythematous plaque was observed consistent with localized granuloma annulare. A skin biopsy confirmed the diagnosis, revealing discrete areas of palisading histiocytes surrounding collections of mucin with perivascular lymphocytes. Based on these findings, a diagnosis of GA was made. Prior treatments included high-potency topical corticosteroids (clobetasol), topical calcineurin inhibitors (tacrolimus), and topical ruxolitinib cream (Opzelura), all of which failed to produce meaningful improvement (Table I).

**Table I:** Prior treatment history for granuloma annulare lesions

Treatment	Formulation	Duration of use	Response
Clobetasol propionate	0.05% cream, BID	∼8 wks	No improvement
Ruxolitinib (Opzelura)	1.5% cream, BID	∼12 wks	No improvement
Tacrolimus	0.1% ointment, QD	∼12 wks	No improvement

Prior treatment history for granuloma annulare lesions in a 66-year-old female. Duration, formulation, and clinical response to each therapy are summarized. All prior topical treatments failed to produce meaningful improvement before initiation of topical ivermectin.

**Fig 1:**
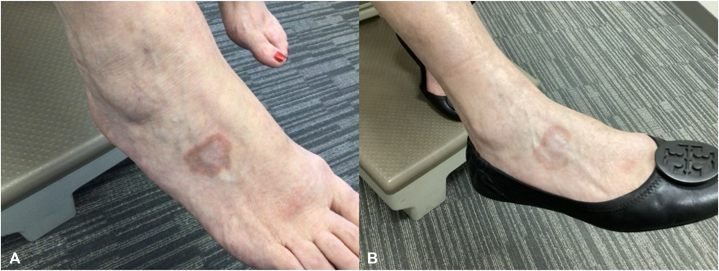
Right dorsal foot of a 66-year-old female with localized granuloma annulare confirmed by biopsy. **A,** Annular, erythematous papules coalescing into plaques prior to treatment. **B,** Significant fading of the lesions after 12 weeks of topical ivermectin 1% cream applied twice daily, demonstrating marked clinical improvement.

**Fig 2:**
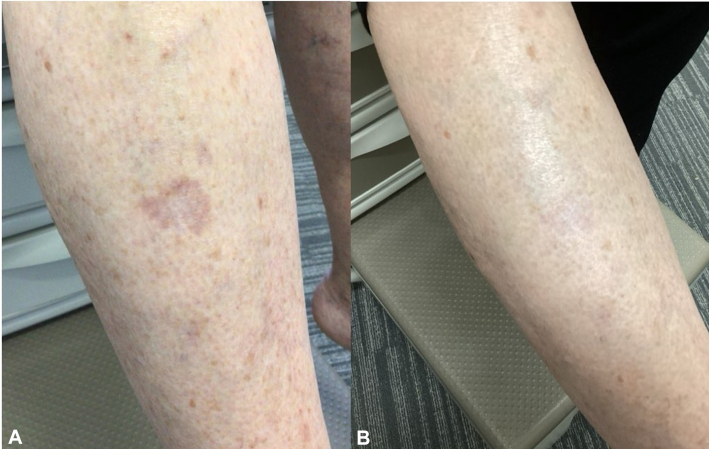
Right calf of a 66-year-old female with localized granuloma annulare confirmed by biopsy. **A,** Annular, erythematous papules coalescing into plaques prior to treatment. **B,** Marked clearance of lesions after 12 weeks of topical ivermectin 1% cream applied twice daily, demonstrating significant clinical improvement.

Considering the persistence of lesions and inadequate response to prior standard topical therapies, the patient was initiated on topical ivermectin 1% cream (Soolantra), applied twice daily for 12 week following a 1-month tacrolimus washout. Remarkably, the GA lesions had made strong improvement by the end of the 12-week course. At her 6-month follow-up visit, she demonstrated significant clinical improvement, with marked fading, clearance of the annular lesions, as well as complete resolution of pruritus (Figs 1 and 2). Physician Global Assessment scores improved from a 3 to a 1 on the right dorsal foot and from a 2 to a 0 on the right calf following treatment, indicating marked clinical improvement and complete clearance of the calf lesion. The GA Severity Index demonstrated a reduction from low–moderate severity at baseline to minimal severity following treatment. The regimen was well tolerated, with no reported local or systemic adverse effects.

## Discussion

Granuloma annulare is often self-limited, but persistent cases can be challenging. Standard topicals and immunosuppressants show variable or transient efficacy. Our patient’s lesions were refractory to multiple therapies, highlighting the need for alternative treatments.

Ivermectin was considered for GA based on its anti-inflammatory properties observed in other dermatologic conditions, such as rosacea. Ivermectin has been shown to downregulate inflammatory mediators, including IL-8, TNF-α, and antimicrobial peptides, although direct evidence of these effects in GA is lacking. Gene expression studies in GA have identified activation of Th2 and JAK/STAT signaling, which suggests that therapies capable of modulating inflammatory cascades might have potential efficacy. While the observed response in our patient is encouraging, it raises the hypothesis that ivermectin’s immunomodulatory effects could contribute to clinical improvement in GA, which will require further investigation.

While this represents a single case, with a limitation of a single follow-up visit 6 month postinitiation, the degree and durability of improvement are notable, particularly given prior treatment failures. Emerging topical therapies include calcineurin inhibitors and JAK inhibitors, with variable efficacy in small studies. Our patient failed both, highlighting the challenge of treatment-resistant GA. Topical ivermectin may offer a novel option, with a favorable safety profile and anti-inflammatory properties. Importantly, ivermectin was well tolerated, with no reported local or systemic adverse effects over 3 month of use. This favorable safety profile may offer an advantage over systemic immunosuppressive agents, which are often considered for recalcitrant GA but carry greater risk. While further studies are needed to directly compare these approaches, the case underscores the potential for ivermectin to serve as an additional therapeutic tool for chronic or refractory GA.

## Conclusion

In summary, we report a case of chronic, treatment-resistant granuloma annulare showing a marked and sustained response to topical ivermectin 1% cream. This outcome highlights the potential of ivermectin as a novel therapeutic option for patients who do not respond to conventional topical therapies, including corticosteroids, calcineurin inhibitors, and JAK inhibitors. Given the favorable safety profile observed in this case, further studies are warranted to evaluate the efficacy, safety, and underlying mechanisms of ivermectin in GA and to determine whether this observation can be replicated in larger patient cohorts.

### Declaration of generative AI and AI-assisted technologies in the writing process

During the preparation of this work, the author(s) used Chat-GPT and Open Evidence in order to improve clarity, refine grammar, and enhance the overall readability of the manuscript. After using this tool/service, the author(s) reviewed and edited the content as needed and take(s) full responsibility for the content of the published article.

## Conflicts of interest

None disclosed.
